# Persistent left superior vena cava: Review of the literature, clinical implications, and relevance of alterations in thoracic central venous anatomy as pertaining to the general principles of central venous access device placement and venography in cancer patients

**DOI:** 10.1186/1477-7819-9-173

**Published:** 2011-12-28

**Authors:** Stephen P Povoski, Hooman Khabiri

**Affiliations:** 1 Division of Surgical Oncology, Department of Surgery, Arthur G. James Cancer Hospital and Richard J. Solove Research Institute and Comprehensive Cancer Center, The Ohio State University Medical Center, Columbus, Ohio, 43210, USA; 2 Section of Interventional Radiology, Department of Radiology, The Ohio State University Medical Center, Columbus, Ohio, 43210, USA

**Keywords:** central venous access, venography, cancer, persistent left superior vena cava, superior vena cava

## Abstract

Persistent left superior vena cava (PLSVC) represents the most common congenital venous anomaly of the thoracic systemic venous return, occurring in 0.3% to 0.5% of individuals in the general population, and in up to 12% of individuals with other documented congential heart abnormalities. In this regard, there is very little in the literature that specifically addresses the potential importance of the incidental finding of PLSVC to surgeons, interventional radiologists, and other physicians actively involved in central venous access device placement in cancer patients. In the current review, we have attempted to comprehensively evaluate the available literature regarding PLSVC. Additionally, we have discussed the clinical implications and relevance of such congenital aberrancies, as well as of treatment-induced or disease-induced alterations in the anatomy of the thoracic central venous system, as they pertain to the general principles of successful placement of central venous access devices in cancer patients. Specifically regarding PLSVC, it is critical to recognize its presence during attempted central venous access device placement and to fully characterize the pattern of cardiac venous return (i.e., to the right atrium or to the left atrium) in any patient suspected of PLSVC prior to initiation of use of their central venous access device.

## Background

Central venous access device placement is a commonplace practice for many physicians, including surgeons, interventional radiologists, and other physicians, who are involved in the management of cancer patients [1]. Yet, successful placement of such central venous access devices can sometimes be very challenging. Therefore, having a thorough understanding of venous anatomy, including the recognition of congenital venous anomalies and the recognition of treatment-induced or disease-induced alterations in thoracic central venous anatomy, as well as having a good working knowledge of alternative and supplemental strategies for placement of central venous access devices, are critical factors to maximizing the success of device placement and to minimizing the risk of potential complications [1-5].

The aim of the current report is to review the available literature as it pertains to the specific congential venous anomaly of the thoracic systemic venous return, persistent left superior vena cava (PLSVC), and to discuss the clinical implications and relevance of congenital aberrancies, as well as of treatment-induced or disease-induced alterations in the anatomy of the thoracic central venous system, as they pertain to the general principles of central venous access device placement and venography. A thorough understanding of such principles is of upmost importance to surgeons, interventional radiologists, and other physicians whom are actively involved in central venous access device placement in cancer patients.

## Case report

The patient was a 53 year old Caucasian woman, without any previous major medical problems, who was recently diagnosed with synchronous bilateral breast cancers and who underwent a right modified radical mastectomy and a left total mastectomy and left axillary sentinel lymph node biopsy for a pT2, pN1, estrogen receptor positive, progesterone receptor positive, HER-2/neu negative invasive lobular carcinoma of the right breast and a pT1b, pN0, estrogen receptor positive, progesterone receptor positive, HER-2/neu negative invasive ductal carcinoma of the left breast, respectively. The patient was subsequently recommended for placement of a subcutaneous implanted port for administration of postoperative adjuvant systemic chemotherapy.

**Figure 1:**
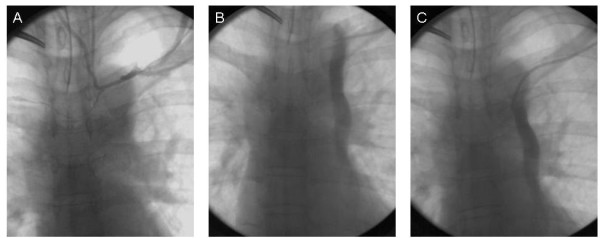
**Intraoperative venography performed by standard fluoroscopy in a non-digital subtraction fashion through a 9.6 French single lumen silicone catheter by way of a left cephalic vein cutdown approach**. (A) Catheter tip is positioned in the region of the mid-portion of the left subclavian vein at a point at which some resistant to further advancement of the catheter was noted. (B) Catheter tip is positioned more centrally, but still horizontally, in the region of the left subclavian vein. (C) Catheter tip is positioned even more centrally and in a craniocaudal direction in the upper left paramediastinal border region.

Therefore, the patient was taken to the operating room by the surgeon for subcutaneous port placement. At the request of the patient, this procedure was done under general anesthesia. The left side was selected, as it represented the side of her earlier-stage breast cancer. A left cephalic vein cutdown approach was undertaken in the left lateral infraclavicular region, by the methodology as previously described by Povoski [4]. Upon creating a transverse venotomy in the anterior wall of the left cephalic vein and passing a 9.6 French single lumen silicone catheter centrally, it was noted on real-time intraoperative fluoroscopy of the thoracic region that the 9.6 French single lumen silicone catheter eventually advanced downward in a craniocaudal fashion along the left paramediastinal border. As a result of this finding, intraoperative venography (Figure 1) was undertaken by the surgeon in a non-digital subtraction fashion through the 9.6 French single lumen silicone catheter and at several distances from the entry point into the left cephalic vein, as the 9.6 French single lumen silicone catheter was sequentially advanced from approximately the 8 cm mark to the 15 cm mark. A total of approximately 50 milliliters of iohexol injectable contrast (300 mg/mL) was utilized during intraoperative venography. With the tip of the 9.6 French single lumen silicone catheter first positioned in the region of the mid-portion of the left subclavian vein, but at a point at which some resistant to further advancement of the 9.6 French single lumen silicone catheter was noted, intraoperative venography performed through the 9.6 French single lumen silicone catheter (Figure 1A) revealed a small (3 to 4 mm) venous branch off of the left subclavian vein that was first directed horizontally for approximately 3 to 4 cm and then was re-directed cephalad in a rightward direction across the upper thorax/lower neck region. Just before the transition from the horizontal to cephalad portion of this small (3 to 4 mm) venous branch off of the left subclavian vein, a tiny (1 to 2 mm) venous tributary was seen to originate off of the small (3 to 4 mm) venous branch. This tiny (1 to 2 mm) venous tributary was noted to meander in a generalized horizontal fashion across the midline of the upper thorax region and into the contralateral right hemi-thorax region. Subsequently, after repositioning of the 9.6 French single lumen silicone catheter and overcoming the previous resistence to catheter advancement, and with the tip of the 9.6 French single lumen silicone catheter now positioned more centrally (but still horizontally) in the region of the left subclavian vein (Figure 1B), and then with further catheter advancement with the tip of the 9.6 French single lumen silicone catheter then positioned even more centrally in a craniocaudal fashion in the upper left paramediastinal border region (Figure 1C), intraoperative venography revealed the presence of a relatively large diameter craniocaudally-oriented venous structure located to the left side of the midline in the medial left hemi-thorax region in a location adjacent to the cardiomediastinal silhouette and which appeared to eventually drain into the cardiac silhouette. There was absence of visualization of an identifiable left innominate vein on intraoperative venography. This relatively large diameter craniocaudally-oriented venous structure coursing downwards on the left side of the midline in the medial left hemi-thorax region was intraoperatively suspected by the surgeon to represent a PLSVC.

Same-day consultation with the interventional radiologist revealed a similar opinion. However, based upon the intrinsic limitations of the non-digital subtraction intraoperative venography procedure performed, an accurate assessment of the point of insertion of the PLSVC into the venous return of the heart and the anatomy of the contralateral right-sided central venous system could not be adequately determined. Therefore, subsequent standard digital subtraction venography was recommended by the interventional radiologist.

The subcutaneous port placement procedure was uneventfully completed by the surgeon by placing the tip of the 9.6 French single lumen silicone catheter to approximately the 15 cm mark within the recognized PLSVC and attaching the 9.6 French single lumen silicone catheter to an implantable port (Titanium Bard PowerPort, C. R. Bard, Inc., Salt Lake City, UT) and closing the port insertion surgical skin incision site that was located in the left lateral infraclavicular region.

**Figure 2:**
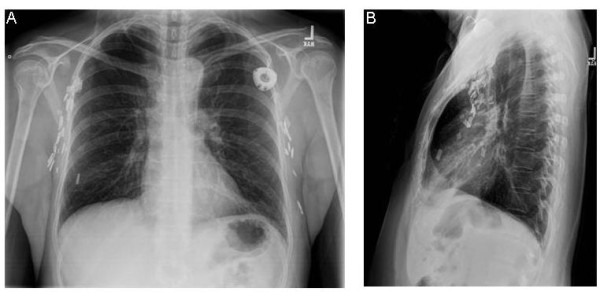
**Posterioranterior (A) and lateral (B) chest x-ray views**.

A subsequent posterioranterior and lateral chest x-ray (Figure 2) was performed and demonstrated the implanted left-sided subcutaneous port and the attached 9.6 French single lumen silicone catheter and its course along the medial left hemi-thorax region in a location adjacent to the cardiomediastinal silhouette, consistent with PLSVC.

**Figure 3:**
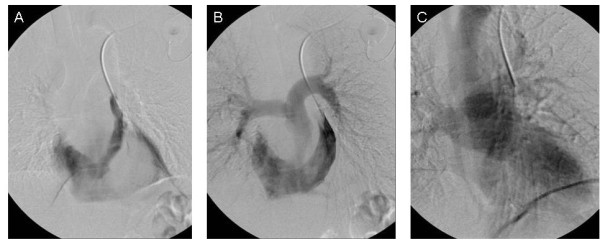
**Digital subtraction venogram of the left-sided central venous system performed by way of the left-sided subcutaneous port**.

**Figure 4:**
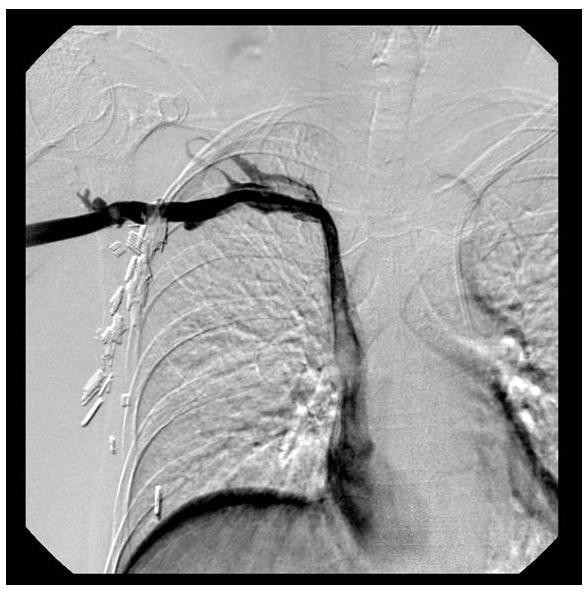
**Digital subtraction venogram of the right upper extremity veins and right-sided central venous system performed by way of a peripheral vein in the dorsum of the right hand**.

In the subsequent weeks after left-sided subcutaneous port placement, digital subtraction venography of the left-sided central venous system (by way of the left-sided subcutaneous port) (Figure 3) and digital subtraction venography of the right upper extremity veins and right-sided central venous system (by way of a peripheral vein in the dorsum of the right hand) (Figure 4) were both performed by the interventional radiologist within the interventional radiology suite.

At approximately two weeks after left-sided subcutaneous port placement, the patient underwent digital subtraction venography of the left-sided central venous system by way of the left-sided subcutaneous port (Figure 3), in order to fully characterize the central venous drainage pathway of the PLSVC (i.e., the point of confluence of the PLSVC with the venous return of the heart). The left-sided subcutaneous port reservoir was accessed in a sterile fashion using an 18-gauge Huber needle. Power injections were performed at 5 mL/second of iodixanol injectable contrast (320 mg/mL), with maximum injection pressure set at 300 PSI. Digital subtraction imaging was performed at 6 frames/second during the power injection. Digital subtraction venography confirmed that the point of confluence of the PLSVC with the venous return of the heart was at the right coronary sinus and into an atrial structure within the cardiac silhouette (Figure 3A). Delayed digital subtraction images demonstrated that the atrial structure then drained into the right ventricle and subsequently into the pulmonary arteries (Figure 3B), confirming that the atrial chamber receiving the venous return from the PLSVC was indeed the right atrium. Further later delayed digital subtraction images demonstrated the pulmonary venous return and the filling of the left side of the heart and subsequent aortic outflow (Figure 3C). There was no evidence of early arterial filling. There was no evidence of right to left shunting on the early images, nor was there evidence of left to right shunting on the delayed images.

Approximately four weeks later, the patient underwent digital subtraction venography of the right upper extremity veins and the right-sided central venous system by way of a peripheral vein in the dorsum of the right hand (Figure 4), in order to fully characterize the right-sided peripheral and central venous anatomy. A vein in the dorsum of the right hand was accessed in a sterile fashion using an 18-gauge angiocatheter. Power injections were performed at 3 mL/second of iodixanol injectable contrast (320 mg/mL), with maximum injection pressure set at 600 PSI. Digital subtraction imaging was performed at 3 frames/second during the power injection. Digital subtraction venography demonstrated normal venous anatomy within the right forearm and right upper arm regions. The more central right-sided veins, including the right axillary vein and right subclavian vein were also normal in appearance. Incidentally, there was partial fenestration of a portion of the right subclavian vein, a commonly encountered venous entity, which is usually of no clinical significance. Her right superior vena cava (SVC) was somewhat smaller in caliber than is usually seen in someone without a co-existing PLSVC. However, her right SVC was approximately of the same size as her PLSVC that was seen on her prior venography imaging. The right SVC venous return to the heart was into the right atrium, and without venographic evidence of right-to-left shunting or left-to-right shunting. The venous flow from the right atrium was identical to that seen during the previous digital subtraction venogram of the left-sided central venous system performed by way of the left-sided subcutaneous port.

Thereafter, the patient was allowed to use her left-sided subcutaneous port for continued administration of postoperative adjuvant systemic chemotherapy, blood draws, and all necessary subsequent contrast-based imaging. The patient had no detectable problems during the utilization of her left-sided subcutaneous port and had no resultant complications. The patient's left-sided subcutaneous port was eventually removed after she completed her postoperative adjuvant systemic chemotherapy, some seven months after its original placement.

## Review

### Origins of the first description and available literature on PLSVC

The exact origin of the first description of PLSVC remain a matter of much great debate within the historical scientific literature, although it appears to have likely occurred at some time during the 17^th ^century to 18^th ^century [6,7]. Some have accredited the recognition of the first description of PLSVC to the work of various individuals during that time period, including the Danish physician Thomas Bartholin (1616-1680) [6-9], the English surgeon William Cheselden (1688-1752) [6,7,10,11], the French surgeon Claude-Nicolas Le Cat (1700-1768) [6,12,13], the Swiss physician Albrecht von Haller (1708-1777) [6,7,14], the German physician Philipp Adolf Boehmer (1711-1789) [6,7,15], and the Swedish surgeon Adolph Murray (1750-1803) [6,7,16,17]. However, the first in-depth review on the topic of the great anterior veins of the thoracic region, including PLSVC, in man and mammals, was published in 1850 by John Marshall (1818-1891), an English surgeon and teacher of anatomy at University College Hospital in London [6].

Since that time, a plethora of, and too numerous to cite, papers have been published on various aspects of PLSVC, including characterization of central venous anatomy and central venous anomalies, embryologic development of the central venous system, identification of PLSVC during implantable pacemaker and cardioverter defibrillator placement, identification of PLSVC during various forms of central venous access device placement, impact of PLSVC on various cardiac surgery procedures, and indications for surgical correction of PLSVC. To date, in PubMed.gov [18], a search of the key words "left superior vena cava" reveals 3109 citations and a search of the key words "persistent left superior vena cava" reveals 923 citations. Some more historical reports on PLSVC [6,7,19-30] and some review-style reports on PLSVC [31-44] are worth mentioning for further reading and have been cited within the current paper.

Those papers in the literature that have specifically addressed the incidental finding of PLSVC at the time of placement of some sort of central venous access device or some sort of central venous monitoring device [38,42,44-123] have generally been directed towards physicians practicing anesthesia [47,48,52,55,58-60,62,64,65,68-71,73,78,85,88,91,92,94,102,117,120,123], critical care [45,46,49-51,53,56,57,72,77,79,83,106,109,110,114,116,119,122], and nephrology [54,66,74,76,80,87,95,96,100,103,104,107,108,113,118]. Despite the fact that a plethora of papers have been published on various aspects of PLSVC and despite there being multiple case reports describing the incidental finding of PLSVC at the time of central venous device placement, there has been very little in the literature specifically directed toward the potential importance of the incidental finding of PLSVC to surgeons, interventional radiologists, and other physicians who are actively involved in central venous access device placement in cancer patients [61,81,82,84,111].

### Incidence of PLSVC

PLSVC represents the most common congenital venous anomaly of the thoracic systemic venous return [36,43]. It is reported to occur in only 0.3% to 0.5% of individuals in the general population, thus representing an occurrence in only 1 in every 200 people to only 1 in every 325 people. However, since the vast majority of cases of this congenital venous anomaly are asymptomatic, its true incidence in the general population may actually be difficult to accurately establish [43]. Nevertheless, it is reported that PLSVC may occur in as many as up to 12% of individuals with other documented congential heart abnormalities [124-126]. The most common associated congential heart abnormalities are atrial septal defect and ventricular septal defect, followed by aortic coarctation, transposition of the great vessels, Tetralogy of Fallot, and anomalous connections of the pulmonary veins [43,124,126]. Conversely, the most frequently associated extra-cardiac anomaly is esophageal atresia [43].

### Anatomic variations of PLSVC

PLSVC can occur in several anatomic variations. Most commonly, PLSVC coexists with a right SVC in up to 80% to 90% of cases [43]. While in many cases, these bilateral SVCs are of relatively equal size, various degrees of size differential can exist between that of the right SVC and the PLSVC [43]. In the instance of bilateral SVCs, a left innominate vein may be completely absent in up to approximately 65% of such cases [42]. In approximately 80% to 92% of cases of PLSVC, the PLSVC [42,43,103] drains into the right atrium via the coronary sinus, resulting in no hemodynamic consequence. Conversely, in approximately 10% to 20% of cases of PLSVC, the PLSVC can drain via the left atrium, either through an unroofed coronary sinus or in a straight line fashion into the roof of the left atrium or through the left superior pulmonary vein [43,44,111]. In the instance of bilateral SVCs, the right SVC generally drains normally into the right atrium [43]. When a PLSVC is identified, the right SVC can be absent in approximately 10% to 20% of cases [23,41,43,44,60,99,121].

### Venous imaging modalities

If it is suspected that a patient has a PLSVC at the time of attempted central venous access device placement, then it is essential for that patient to undergo subsequent appropriate investigations to fully characterize their central venous anatomy. This is important in order to confirm the presence of PLSVC, to characterize the central venous anatomy of the contralateral right side, to characterize the pattern of cardiac venous return to the right atrium or to the left atrium, and to evaluate the patient for other potential coexisting congential heart abnormalities. Multiple venous imaging modalities can be utilized, as well as used in concert with one another, to accomplish complete characterization of the central venous anatomy. These venous imaging modalities include conventional contrast venography, transthoracic echocardiography, transesophageal echocardiography, multidetector computed tomography venography, and magnetic resonance venography [2,3,41,89,127-134]. Conventional contrast venography can be performed in the operating room (most commonly available by using single-image, non-digital subtraction intraoperative fluoroscopy techniques and less commonly available by digital subtraction intraoperative venography) or in the interventional radiology suite (generally always available by digital subtraction venography). Along similar lines, these venous imaging modalities can be utilized upfront prior to attempted central venous access device placement, if a patient is suspected of having pre-existing treatment-induced or disease-induced alterations in central venous anatomy.

### Clinical relevance of PLSVC to central venous access device placement

As previously mentioned, the incidental finding of a PLSVC during central venous access device placement is of great potential importance to surgeons, interventional radiologists, and other physicians who are actively involved in central venous access device placement in cancer patients. With PLSVC occurring in only 0.3% to 0.5% of individuals in the general population and since there is theoretically only a 50% chance of encountering a PLSVC in an individual who has a PLSVC (by assuming that 50% of PLSVCs would be missed by a physician selecting the right side instead of the left side as the site of insertion of any given central venous access device), then it is very plausible that most physicians who place central venous access devices in their clinical practice may possible never, or only once, come across this congenital venous anomaly during their careers. In this regard, a resultant patient outcome in this rarely encountered scenario could potentially be devastating if the possibility of PLSVC was not thought of and/or not recognized by the physician at the time of a "difficult" central venous access device placement procedure.

It is important to discuss the implications of PLSVC as it applies to the pattern of cardiac venous return (i.e., to the right atrium or to the left atrium) in any given patient suspected of PLSVC at central venous access device placement. As previously discussed, the venous return from the PLSVC drains into the left atrium in approximately 10% to 20% of cases [43,44,111]. This particular venous drainage pattern of PLSVC that results in venous return to the left atrium [34,40,43,44,65,81,92,98,100,111,113], as well as any other cardiac anomaly which results in right-to-left cardiac shunting, places those patients at a significant risk for subsequent paradoxical embolic complications to the arterial system, either from thromboemboli or air emboli, with resultant neurologic, cardiac, renal, mesenteric, and/or peripheral sequelae [34,40,44,65,81,92,98,100,111,113,135,136]. Therefore, it is essential that one fully characterizes, by venous imaging, the pattern of cardiac venous return (i.e., to the right atrium or to the left atrium) in any patient suspected of PLSVC at central venous access device placement prior to initiation of use of their central venous access device.

### Clinical indications for and relevance of venography during selected cases of attempted central venous access device placement: the surgeon's perspective and the interventional radiologist's perspective

From the surgeon's perspective, intraoperative venography during attempted central venous access device placement can be a very useful tool for immediate characterization of central venous anatomy, including for recognition of congenital venous anomalies such as PLSVC, as well as for recognition of treatment-induced or disease-induced alterations in thoracic central venous anatomy. The use of intraoperative venography techniques during attempted central venous access device placement has been previously discussed in detail, from the surgeon's perspective, by one of the present authors for the venous cutdown approach and for the percutaneous venipuncture approach in cancer patients [2,3], as well as has been previously discussed by other authors for the percutaneous venipuncture approach in hemodialysis patients [137]. Its use during attempted central venous access device placement should be considered in selected cases, such as in those instances in which there is difficulty with passing/advancing the guidewire or the central venous access catheter and in those instances in which aberrant catheter position is suspected [2,3]. In most operating room suites, intraoperative venography is generally performed using single-image, non-digital subtraction intraoperative fluoroscopy techniques, unless such an operating room suite is equipped with specialized digital subtraction fluoroscopy equipment.

From the surgeon's perspective, an important point of discussion regarding the performance of intraoperative venography during attempted central venous access device placement relates to the method of venous access (i.e., percutaneous venipuncture approach versus venous cutdown approach) and to the stepwise timing of intraoperative venography during attempted central venous access device placement [2,3]. In this regard, it should be clearly noted that the vast majority of surgeons still utilize the percutaneous venipuncture approach to either the left subclavian vein or right subclavian vein, with far fewer using the percutaneous venipuncture approach to either the right internal jugular vein or left internal jugular vein. Only a minority of surgeons utilize a venous cutdown approach, generally to either of the cephalic veins or external jugular veins, for central venous access device placement. Although readily available and endorsed by the American College of Surgeons [138], the vast majority of surgeons performing a percutaneous venipuncture approach to the subclavian vein or internal jugular vein still do not routinely utilize venous ultrasound to guide the placement of the venipuncture needle into the initial point of entry into the selected venous structure at the time of attempted central venous access device placement.

Performance of intraoperative venography by the surgeon via a venous cutdown approach (i.e., cephalic vein approach or external jugular vein approach) at the time of attempted central venous access device placement represents a very safe, straightforward, and highly useful means for obtaining detailed intraoperative characterization of the central venous anatomy [2,3]. By injecting contrast into the central venous access catheter with its tip located at the point of entry into the most peripheral venous conduit (i.e., cephalic vein or external jugular vein), and then sequentially advancing the catheter centrally, one can obtain a relatively detailed venous roadmap of the ipsilateral subclavian vein, innominate vein, and SVC, even when only single-image, non-digital subtraction intraoperative fluoroscopy techniques are available and employed.

On the other hand, performance of intraoperative venography by the surgeon via a percutaneous venipuncture approach (i.e., percutaneous subclavian vein approach or percutaneous internal jugular vein approach) at the time of attempted central venous access device placement has some intrinsic limitations [2,3]. Although the percutaneous venipuncture approach to central venous assess can also allow for the injection of contrast at the initial point of entry into the most peripheral venous conduit (i.e., subclavian vein or internal jugular vein), it is only possible during the early phases of the modified Seldinger technique when the venipuncture needle or an equivalent-sized dilator is still in place or even as far into the procedure as when the dilator and peel-away sheath apparatus are still in place (with or without the insertion of the central venous assess catheter). However, once the central venous access catheter has been passed through the peel-away sheath and advanced to its anticipated final central venous location at the junction of the SVC and right atrium, and the peel-away sheath has been subsequently peeled back off from the catheter, then intraoperative venography, in a practical sense, can only be performed through the tip of the already centrally placed catheter. At any time prior to peeling back the peel-away sheath, the central venous assess catheter and surrounding intact peel-away sheath can, to some degree, be manipulated and drawn back more peripherally for attempting intraoperative venography through the catheter tip positioned within a more peripheral portion of the central veins. Obviously, however, once the final positioning of the tip of the central venous access catheter is determined within the presumed most ideal location within the SVC region and is set by the process of peeling back the peel-away sheath during the modified Seldinger technique, then, if one attempts intraoperative venography, one simply tends to see only rapid contrast dissipation (i.e., washout) and the inability to obtain readable intraoperative fluoroscopic images when using single-image, non-digital subtraction intraoperative fluoroscopy techniques. As previously discussed elsewhere [2], this very straightforward concept regarding the importance of catheter tip position within the central venous system and the practicality of performing intraoperative venography that will yield readable intraoperative fluoroscopic images has formerly failed to be recognized by surgeons and other physicians alike whom are involved in central venous access device placement in cancer patients [139]. However, in this particular instance, the availability of digital subtraction intraoperative fluoroscopy equipment in the operating room may provide the surgeon with an increased opportunity and likelihood for obtaining better intraoperative fluoroscopic images for attempting to possible define any central venous aberrancies. Nevertheless, such specialized digital subtraction intraoperative fluoroscopy equipment is rarely available to surgeons in most operating room suites, and for the most-part, the majority of operating room suites are still equipped with single-image, non-digital subtraction intraoperative fluoroscopy technology.

From the interventional radiologist's perspective, the approach to central venous access device placement and to venography is somewhat different than the surgeon's approach, as it relates to the method of venous access, the vein selection site, and the method of venography. In this regard, whereas surgeons primarily utilize the left subclavian vein or right subclavian vein percutaneous venipuncture approach (more commonly without ultrasound guidance), interventional radiologists almost exclusively utilize an ultrasound-guided right internal jugular vein percutaneous venipuncture approach, and alternatively an ultrasound-guided left internal jugular vein percutaneous venipuncture approach when there is a contraindication to central venous access device placement on the right side. After initial successful placement of the venipuncture needle by the ultrasound-guided right internal jugular vein or left internal jugular vein percutaneous venipuncture approach, the interventional radiologist passes the guidewire and watch the guidewire pass down through the thorax region under fluoroscopy and use the course of the guidewire and its behavior within the central veins as reasonable validation of standard/normal central venous anatomy. If there is any suspicious behavior by the guidewire (i.e., failure to advance the guidewire centrally into the SVC, or having the guidewire take a non-standard route), then the venipuncture needle is generally removed over the guidewire, a 5-French dilator is passed over the guidewire, the guidewire is then removed, and a venogram is performed. Injection of contrast into the 5-French dilator at this very peripheral initial point of entry into the right internal jugular vein or left internal jugular vein by the interventional radiologist will again allow for a relatively detailed venous roadmap of the ipsilateral internal jugular vein, subclavian vein, innominate vein, and SVC using digital subtraction intraoperative fluoroscopy equipment that is routinely available in the interventional radiology suite. Such an approach by the interventional radiologists has been developed out of necessity and in response to the increasing number of patients that are encountered with treatment-induced or disease-induced alterations in thoracic central venous anatomy.

### Clinical indications for and relevance of venous ultrasound during attempted central venous access device placement by way of the percutaneous venipuncture approach: the surgeon's perspective and the interventional radiologist's perspective

It is well-established within the radiology [140], anesthesia [141], and surgical [138,142] literature that venous ultrasound is a very useful and recommended imaging tool for guiding successful placement of the venipuncture needle into the initial point of entry of the selected venous structure, such as the subclavian vein or internal jugular vein, during the percutaneous venipuncture approach to central venous access device placement. While interventional radiologists have fairly universally embraced the use of venous ultrasound to help successfully guide the placement of the venipuncture needle into the initial point of entry of the selected venous structure during the percutaneous venipuncture approach to central venous access device placement in the interventional radiology suite, surgeons have been far more resistant to incorporating venous ultrasound into their repertoire for central venous access device placement in the operating room. Despite the proven usefulness of venous ultrasound for guiding successful placement of the venipuncture needle into the initial point of entry of the selected venous structure during the percutaneous venipuncture approach to central venous access device placement [138,140-142], it is nevertheless well recognized that venous ultrasound that is performed to the proximal upper extremity veins and central veins of the chest region can actually miss up to 50% of venous abnormalities that are otherwise clearly identifiable on conventional contrast venography [2,3,137,143-147], including on intraoperative venography [2,3,137]. This is most easily explainable by the fact that many venous abnormalities of the upper extremity and central veins of the chest region are located in a more central location within the thoracic venous system (i.e., along the medial segment of the subclavian vein, along the innominate vein, or within the SVC), thus representing more centrally-located segments of the thoracic central venous anatomy which are not ideally accessible for visualization by standard venous ultrasound techniques [2,3]. Thus, from the surgeon's perspective, independent of whether or not one chooses to utilize venous ultrasound to guide the initial point of entry into the selected venous structure during the percutaneous venipuncture approach to central venous access device placement, the utilization of venography at the time of attempted central venous access device placement, by either a venous cutdown approach or a percutaneous venipuncture approach, can be an invaluable tool for defining the central venous anatomy and for providing a venous roadmap in particularly challenging cases in which difficulties are encountered during attempted central venous access device placement [2,3,137].

## Conclusions

A thorough understanding of venous anatomy, including the recognition of congenital venous anomalies (such as PLSVC) and the recognition of treatment-induced or disease-induced alterations in thoracic central venous anatomy, as well as having a good working knowledge of alternative and supplemental strategies for placement central venous access devices, are all critical factors to maximizing the success of central venous access device placement and to minimizing the risk of potential complications. A thorough understanding of these principles is of upmost importance to surgeons, interventional radiologists, and other physicians whom are actively involved in central venous access device placement in cancer patients.

Specifically regarding PLSVC, it is critical to recognize its presence during attempted central venous access device placement and to fully characterize the pattern of cardiac venous return (i.e., to the right atrium or to the left atrium) in any patient suspected of PLSVC prior to initiation of use of their central venous access device.

## Abbreviations

PLSVC: persistent left superior vena cava; SVC: superior vena cava

## Consent

Written informed consent was obtained from the patient for publication of this review paper and accompanying images. A signed copy of the written consent form from the patient is available for review by the Editor-in-Chief of this journal.

## Competing interests

The authors declare that they have no competing interests.

## Authors' contributions

**SPP **was the surgeon who performed the central venous access device placement procedure and the intraoperative venogram procedure. **HK **was the interventional radiologist who performed the postoperative venogram procedures. Both of the authors were involved in writing and editing this manuscript. Both of the authors have read and approved the final version of this manuscript.
